# Expression of naturally ionic liquid-tolerant thermophilic cellulases in *Aspergillus niger*

**DOI:** 10.1371/journal.pone.0189604

**Published:** 2017-12-27

**Authors:** Saori Amaike Campen, Jed Lynn, Stephanie J. Sibert, Sneha Srikrishnan, Pallavi Phatale, Taya Feldman, Joel M. Guenther, Jennifer Hiras, Yvette Thuy An Tran, Steven W. Singer, Paul D. Adams, Kenneth L. Sale, Blake A. Simmons, Scott E. Baker, Jon K. Magnuson, John M. Gladden

**Affiliations:** 1 Joint BioEnergy Institute (JBEI), Biological Systems & Engineering Division, Lawrence Berkeley National Laboratory, California, United States of America; 2 Chemical and Biological Processes Development Group, Pacific Northwest National Laboratory, Richland, Washington, United States of America; 3 Biomass Science and Conversion Technologies Department, Sandia National Laboratories, Livermore, California, United States of America; 4 Department of Geochemistry & Department of Ecology, Earth Sciences Division, Lawrence Berkeley National Laboratory, Berkeley, California, United States of America; 5 Department of Molecular and Cell Biology, University of California-Berkeley, Berkeley, California, United States of America; 6 Environmental Molecular Sciences Laboratory, Richland, Washington, United States of America; University of Nottingham, UNITED KINGDOM

## Abstract

Efficient deconstruction of plant biomass is a major barrier to the development of viable lignocellulosic biofuels. Pretreatment with ionic liquids reduces lignocellulose recalcitrance to enzymatic hydrolysis, increasing yields of sugars for conversion into biofuels. However, commercial cellulases are not compatible with many ionic liquids, necessitating extensive water washing of pretreated biomass prior to hydrolysis. To circumvent this issue, previous research has demonstrated that several thermophilic bacterial cellulases can efficiently deconstruct lignocellulose in the presence of the ionic liquid, 1-ethyl-3-methylimadizolium acetate. As promising as these enzymes are, they would need to be produced at high titer in an industrial enzyme production host before they could be considered a viable alternative to current commercial cellulases. *Aspergillus niger* has been used to produce high titers of secreted enzymes in industry and therefore, we assessed the potential of this organism to be used as an expression host for these ionic liquid-tolerant cellulases. We demonstrated that 29 of these cellulases were expressed at detectable levels in a wild-type strain of *A*. *niger*, indicating a basic level of compatibility and potential to be produced at high levels in a host engineered to produce high titers of enzymes. We then profiled one of these enzymes in detail, the β-glucosidase A5IL97, and compared versions expressed in both *A*. *niger* and *Escherichia coli*. This comparison revealed the enzymatic activity of A5IL97 purified from *E*. *coli* and *A*. *niger* is equivalent, suggesting that *A*. *niger* could be an excellent enzyme production host for enzymes originally characterized in *E*. *coli*, facilitating the transition from the laboratory to industry.

## Introduction

Lignocellulosic biofuels overcome some of the major issues surrounding first-generation biofuels produced from food crops, such as corn, and have great potential to reduce both petroleum-based fuel dependency and environmental concerns related to fossil CO_2_ emissions. Lignocellulose, an abundant and widely available renewable carbon source, is primarily composed of 40–50% cellulose, 25–30% hemicellulose, and 15–20% lignin [1,2]. Technoeconomic analysis has indicated that conversion of C5 and C6 sugars derived from lignocellulose into biofuels is a less expensive and more sustainable route to produce biofuels compared to current starch-based technologies [1]. These sugars can be liberated from lignocellulose through enzymatic hydrolysis of cellulose and hemicellulose using cellulases and hemicellulases, respectively [3,4]. However, these glycoside hydrolases are the major cost drivers of the deconstruction process, and therefore development of technologies that reduce enzyme costs will be critical for the successful commercialization of lignocellulosic biofuels [5]. Accordingly, the development of novel deconstruction technologies that reduce enzyme costs has been a major focus area in the lignocellulosic biofuels research community [6].

One significant route to decreasing enzyme costs is through efficient pretreatment of the biomass to reduce the recalcitrance to enzymatic degradation. Several lignocellulose pretreatment technologies have been developed, and those using ionic liquids (ILs) show great promise. ILs are non-volatile solvents that dissolve various compounds, including cellulose [7]. Lignocellulosic biomass treated with ILs has been shown to be highly susceptible to enzymatic depolymerization and the hydrolysates generated from this process have been used for biofuel production [8].

To implement efficient pretreatment technologies, another route to decreasing costs within a biorefinery is to consolidate unit operations. In that regard, a single unit or one-pot lignocellulose IL-pretreatment and saccharification process has been reported. This approach eliminates water washing steps prior to saccharification, and is estimated to reduce both waste and costs within a biorefinery [9]. The technology hinges on the use of a thermophilic and IL-tolerant cellulase mixture composed of three different classes of cellulases, a β-glucosidase (EC 3.2.1.21), a cellobiohydrolase (CBH; EC 3.2.1.91), and a mixture of endoglucanases (EC 3.2.1.4). The enzymes are active at high temperatures and tolerate up to 15–20% (v/v) of the IL, 1-ethyl-3-methylimadizolium acetate ([C_2_C_1_lm][OAc]), used for pretreatment. For comparison, both individual cellulases and commercial cellulase mixtures derived from filamentous fungi such as *Trichoderma reesei* exhibit much lower activity in the presence of this IL and are almost completely inhibited at IL concentrations as low as 5% (v/v) [10–12].

In addition to the enzymes in in the aforementioned cellulase mixture, another 37 cellulases were isolated from a thermophilic microbial community that exhibited significant IL-tolerance [13]. These thermostable and IL-tolerant enzymes have the potential to be used in a number of biomass conversion technologies that rely on high temperatures and/or ILs. However, in order to develop these technologies, these enzymes need to be produced in a high titer in an industrial production host. Filamentous fungi have the capacity to produce high titers of enzymes, and several have been used in industry to produce enzymes such as glucoamylase and the aforementioned cellulase mixtures. They also secrete enzymes extracellularly, which simplifies the downstream purification process. Altogether, these characteristics make filamentous fungi ideal enzyme production platform for these IL-tolerant cellulases [14].

Several fungi, including *Neurospora*, *Penicillium*, *Trichoderma*, and *Aspergillus* species have been used for cellulase production [1,15]. *A*. *niger*, an ascomycete filamentous fungus, is known to produce a wide range of enzymes, including biomass degrading enzymes. Moreover, some commercial cellulase cocktails are already being produced by *A*. *niger* [16,17]. A variety of molecular biological tools have been developed in *A*. *niger*, thus facilitating fungal transformation and integration of DNA constructs designed for heterologous protein expression [18,19]. In addition, this fungus has been used to produce heterologous proteins, including not only fungal cellulases and hemicellulases [15], but also humanized immunoglobulin G1 (IgG1) [20]. Therefore, *A*. *niger* was chosen in this study to examine its potential as heterologous enzyme expression host. To establish a baseline for heterologous enzyme production, a wild-type strain of *A*. *niger* was selected. A total of 32 genes encoding three different classes of thermophilic cellulases sourced from several different bacteria and fungi were expressed in *A*. *niger* and their enzymatic activity was evaluated. Most of the enzymes expressed well in *A*. *niger*, with the β-glucosidase A5IL97 (UniProt ID)[12] producing the highest detected activity. To further examine heterologous enzyme production in *A*. *niger*, A5IL97 was characterized in more detail. The enzyme was expressed in two hosts, *A*. *niger* and *E*. *coli*, and its enzymatic activity, IL-tolerance and thermal stability were compared. In addition, the *A*. *niger* and *E*. *coli* expressed A5IL97 enzymes were further tested within the context of a cellulase mixture and results indicate that the A5IL97 enzyme is functionally equivalent when produced in either organism. These results suggest that *A*. *niger* is a good potential enzyme expression host for heterologous cellulase enzymes, and lay the initial groundwork for future efforts to engineer high enzyme titer strains for industrial applications.

## Materials and methods

### Heterologous enzyme *A*. *niger* expression construct

A shuttle expression vector, pCB1004-glaA, was built by cloning the sequence containing 846bp of the *A*. *niger* glucoamylase (*glaA)* promoter, signal sequence, pro-peptide, a cloning spacer for insertion of cellulase genes that includes the sequence for a Strep/His dual C-terminal tag, and 574bp of the *A*. *nidulans trpC* terminator sequence into the *Xho*I and *Not*I sites of pCB1004 (S1 Fig). Detailed sequence information is listed in S2 Fig. The pCB1004 vector has both hygromycin and chloramphenicol resistance genes for selection in *A*. *niger* and *E*. *coli*, respectively [21]. The open reading frame (ORF) of each cellulase gene was codon optimized for *A*. *niger*, synthesized (GenScript, Piscataway, NJ), and then cloned into the *Sal*I and *Xba*I sites of the pCB1004-*glaA* vector. These ORFs were inserted upstream and in frame with the sequence for the Strep and 8 × His-tags, to allow the proteins to be detected and purified from the native *A*. *niger* proteins (S1 Fig). Several control genes were included: green fluorescent protein (GFP), and three fungal cellulases from *Thermoascus aurantiacus* and *T*. *reesei* (Table 1).

**Table 1:** Bacterial and fungal cellulases expressed in *A*. *niger*.

Gene ID	Predicted Function	GH	IL-tolerant	Thermo-tolerant	BG	CBH	EG	NCBI GenBank
GFP F64L S65T	control	-	N/A	N/A	<1.0	<1.0	<30.0	KY014123
A5IL97	BG	GH1	Y	Y	2384.0	938.3		KY014108
BGL1	BG	GH3	N/A	N/A	3.0			KY014119
CBH1	CBH	GH7-CBM1	N/A	N/A		3.4	159.0	KY014120
EG1	EG	GH5	N/A	N/A		7.2	76.5	KY014122
Csac	EG	CBM3-GH5	Y	Y			664.2	KY014107
PhoEG	EG	GH5	Y	Y			61.6	KY014117
Prumi	EG	GH26-GH5	N/A	N/A		2.9	126.0	KY014118
Cel5A	EG	GH5	Y	Y		7.7	258.0	KY014121
Cel9A	EG	GH9	N/A	Y		2.6	999.0	KY014109
J01	BG	GH3	Y	N	6.5			KY014124
J02	BG	GH3	Y	Y	25.5			KY014125
J03	BG	GH3	Y	Y	13.5			KY014126
J06	BG	GH1	N	Y	38.9			KY014110
J07	BG	GH1	Y	Y	120.4			KY014111
J08	BG	GH1	Y	Y	1.6			KY014127
J09	BG	GH1	Y	Y	2.2			KY014128
J11	BG	GH3	Y	Y	Negative			KY014129
J14	BG	GH3	Y	Y	158.7			KY014112
J15	BG	GH3	N	Y	14.6			KY014130
J16	BG	GH3	Y	Y	13.5			KY014131
J17	BG	GH3	N	Y	34.6			KY014132
J18	BG	GH1	Y	Y	11.4			KY014133
J19	BG	GH1	Y	Y	13.5			KY014134
JMB19063; J20	BG	GH3	N/A	N/A	140.3			KY014113
J24	EG	GH9	Y	N		2.3	225.0	KY014135
J26	EG	GH12	Y	Y		3.4	420.1	KY014114
J28	EG	GH5	N/A	N/A		2.9	175.5	KY014136
J29	EG	GH5	Y	Y		2.3	249.8	KY014137
J30	EG	GH9	Y	Y		73.6	798.9	KY014115
J31	EG	GH5	N/A	N/A		0.7	126.0	KY014138
J35	EG	GH5	N/A	N/A			134.3	KY014139
J36	EG	GH5	Y	Y		36.4	623.1	KY014116

Glycoside hydrolase (GH) family is categorized based on the CAZy, carbohydrate-active enzyme database (http://www.cazy.org). The organism of origin for each cellulase was predicted by metagenomic binning [30] and described in [13]. BG, CBH, and EG, are β-glucosidase, cellobiohydrolase, and endoglucanase, respectively. IL-tolerant is defined as retaining >50% enzymatic activity in at least 10% (v/v) [C2mim][OAc]. Thermo-tolerant is defined by an optimum temperature for enzymatic activity of greater than 60°C. Data on Thermo- and IL- tolerance was obtained from references [11,13,31]. Enzyme activity numbers represent the highest activity of each plasmid transformant in mU per mL. The experiment was repeated with 4 biological replicates. The GFP strain was used to measure the fungal wild-type level of enzyme activity to calculate the bacterial enzyme activity in the fungal host; less than 1 mU per mL for β-glucosidases and cellobiohydrolase and less than 30 mU per mL for endoglucanase activity at different temperatures. The value was subtracted from the GFP control. Dark grey shading indicates not tested.

### Fungal transformation and culture conditions

*A*. *niger* ATCC® 11414™ was obtained from the American Type Culture Collection (Manassas, VA) and used as the enzyme production host strain for this study. Expression vector plasmid DNA was extracted from *E*. *coli* using the Plasmid Midi Kit (Qiagen, Valencia, CA) and transformed into *A*. *niger* using the polyethylene glycol protocol [22]. Briefly, a 1 L flask containing 250 ml of yeast peptone dextrose broth media was inoculated with 5 × 10^7^ *A*. *niger* spores and shaken at 30°C for 10 hours until the spores germinated. The germlings were then protoplasted following the polyethylene glycol protocol. At least 10 micrograms of each cellulase plasmid was used to transform *A*. *niger*. All fungal mutants were screened and maintained in glycerol stocks and grown for spore collection on potato dextrose agar or minimal media at 30°C with 50% relative humidity. At least 20 independent transformants of each construct used in this study were screened for the integration of the expression construct (S1 Fig) using PCR with SAC001 (5’- GCATCATTACACCTCAGCAATG -3’), and SAC004 primers (5’- CAGTTCGAGAAGGCCGCTC -3’), which amplify the enzyme gene insert from the vector (S1 Fig). The integration was further confirmed by Southern blot with a probe, generated with SAC035 (5’- GCGGCAGTTGATGAATTTCGG -3’) and SAC004 primers (S3 Fig).

For liquid cultures, 50 microliters of freshly collected fungal mutant spores were inoculated into 5 ml of corn steep liquor-fructose media. The cultures were incubated at 37° C at 200 rpm for 24 hours, and then, 500 microliters of the fungal biomass culture was transferred into 5 ml of Promosoy special media [20] with modifications as follows, Promosoy 100 was substituted with 45 g per L Bacto™ Peptone (BD Biosciences, San Jose CA). Antifoam 204 was added to the liquid fungal cultures at 0.05 mL/L (Sigma, St. Louis, MO) instead of Mazu DF60-P (Mazur Chemicals, Gurnee, IL). The liquid cultures were incubated at 30°C, 200 rpm for 72 hours, and the supernatant was collected through a 0.45 um filter (VWR, Radnor, PA). The supernatant was used for cellulase activity assays and protein purification.

### Cellulase activity assays

The β-glucosidase and cellobiohydrolase enzymatic assays were performed with 1 mM of *p*-nitrophenyl-β-D-glucopyranoside (*p*NPG, Sigma, St. Louis, MO) and 5 mM of *p*-nitrophenyl-β-D-cellobioside (*p*NPC, Sigma, St. Louis MO), respectively. The substrates were mixed with 10 μL fungal supernatant in 100 mM MES buffer at pH 6.5, and incubated for 30 minutes at the optimum temperature for each enzyme [13]. Following incubation, an equal volume of 2% Na_2_CO_3_ was added to stop the reaction. The liberated *p*-nitrophenol was detected by its absorbance at 410 nm (Molecular Devices, Sunnyvale CA). Azo-CM-Cellulose (Megazyme, Wicklow, Ireland) was used to measure endoglucanase activity. The manufacturer’s instructions were modified as follows. The Azo-CM-Cellulose substrate was incubated with an equal volume of fungal supernatant in 125 mM MES buffer at pH 6.5. Water was substituted for the fungal supernatant in the negative control. The mixture was incubated at the optimum temperature of each enzyme, and the reaction was quenched with 2.5 volumes of precipitant solution. Liberated azo dye modified oligosaccharide levels in the aqueous layer were measured by absorbance at 590 nm (Molecular Devices, Sunnyvale CA). The enzyme activity unit conversion was calculated, following the Azo-CM-Cellulose manufacturer’s instructions for endoglucanase and following the formula, described by [23] for β-glucosidase and cellobiohydrolase. All enzymatic activity was compared relative to a GFP expressing negative control strain. The GFP strain showed that the wild-type level of enzyme activity was less than 1 mU per mL for β-glucosidases and cellobiohydrolase and less than 30 mU per mL for endoglucanase at different temperatures.

### Protein expression and purification

To purify A5IL97 produced in *A*. *niger*, cultures were scaled up to 50 mL. After growth for 7 days the supernatant was separated from the fungal biomass and 70% of (NH_4_)_2_SO_4_ was added to the supernatant and mixed at 20°C and 200 rpm for 1 hour [24]. The mixture was centrifuged at 10,000 rpm at 4°C for 20 minutes to precipitate the recombinant protein. The pellets were resuspended with 1 × PBS (20 mM Na_2_HPO_4_, 300 mM NaCl) at pH 7.4, and extra salts were removed using a PD-10 desalting column (GE Health Life Sciences, Pittsburgh, PA). Eluted His (×8)-tagged recombinant proteins were purified with HisPur Ni-NTA resin, following the manufacturer’s instructions (Thermo Fisher Scientific, Waltham, MA).

Bacterial recombinant proteins were purified using a previously described method with modifications of the culture conditions [12]. Briefly, A5IL97 was cloned with V5 epitope and His (6×) tags into pDEST42 vector (Thermo Fisher Scientific, Waltham, MA) and expressed in BL21 Star™ (DE3) (Thermo Fisher Scientific, Waltham, MA). One milliliter overnight cultures were transferred to 50 mL of Terrific Broth media, followed by addition of 2 g per L glycerol and 2 mM magnesium sulfate and incubation at 37°C, 200 rpm until reaching 0.6 at OD_600_. Then 500 μM of isopropyl-β-D-thiogalactopyranoside (IPTG) was added and the incubation was continued overnight at 20°C. The cells were collected by centrifugation at 10,000 rpm for 15 minutes at 4°C and then resuspended in BugBuster^®^ with Lysonase™ and Benzonase^®^ (EMD Millipore, Billerica, MA). A5IL97 protein was purified using HisPur Ni-NTA, following manufacturer’s instructions (Thermo Fisher Scientific, Waltham, MA). The collected fractions were analyzed using sodium dodecyl sulphate (SDS)-polyacrylamide gel electrophoresis (PAGE) and enzymatic activity assay, and the protein concentration was determined by the Bradford assay (Bio-Rad, Hercules, CA) (S4 Fig).

### Enzyme activity profiles

#### i) zymogram

Zymography was performed to compare β-glucosidase and cellobiohydrolase activity of A5IL97 expressed in *E*. *coli* and *A*. *niger*. Unpurified recombinant proteins were not boiled and loaded with Novex^®^ Tris-Glycine native sample buffer (2×; Thermo Fisher Scientific, Waltham, MA) into native PAGE gel without SDS. Following electrophoresis, the gels were incubated with 2.5% (v/v) of Triton-X for 1 hour, and then, further incubated with 50 mM sodium citrate, pH 5.0, containing 0.001% of 4-methylumbelliferyl-β-D-glucopyranoside (MUG; Sigma, St. Louis, MO) or 4-methylumbelliferyl-β-D-cellobioside (MUC; Sigma, St. Louis, MO) at 70°C for 10 minutes [25]. The gel was visualized under ultraviolet light then stained with Coomassie Blue G-250 using the manufacturer’s instructions (Thermo Fisher Scientific, Waltham, MA). The zymography assay was repeated at least two times.

#### ii) jSALSA

Enzymatic activity profiles of A5IL97 in *E*. *coli* and *A*. *niger* were generated using jSALSA, the JBEI suite for automated lignocellulosic saccharification. jSALSA is an automated robotic platform established on Biomek FX^P^ and NX^P^ Lab Automation Workstations (Beckman Coulter, Carlsbad, CA) to generate high-throughput cellulase activity profiles over a range of conditions [26]. The enzyme activity profiles were measured, based on six different temperatures (70°C to 95°C, in 5°C increments), four different pHs (3.8, 5.6, 6.6, and 7.8), and four different concentrations of the IL, 1-ethyl-3-methylimidazolium acetate (5%, 10%, 20%, and 30%). Approximately 15 μg/mL of purified recombinant enzyme A5IL97 from *E*. *coli* and *A*. *niger* were used. The buffers for pH were composed of mixtures of low pH (150 mM acetic acid and 50 mM citric acid) and high pH (50 mM sodium citrate tribasic and 100 mM Na_2_HPO_4_) buffers to achieve the desired pH. Ionic liquids with different concentrations were prepared with a pH 5.6 buffer and diluted from concentrated stocks to the appropriate final concentration in 100 μL total reaction volume. The substrates for A5IL97, 1 mM of *p*NPG, or *p*NPC in pH 5.6 buffer were diluted from 5 × stocks in 100 μL total reaction volume. The components were loaded on a 96 well-plate, dispensed, and incubated for 30 min. The reaction was quenched with a half volume of 1.3% Na_2_CO_3_, and the end-product was measured by absorbance at 410 nm. The enzyme activity data were analyzed using MATLAB^®^ software (MathWorks, Natick, MA). Enzymatic activity profiles using jSALSA were repeated at least three times.

#### iii) Thermal stability

Enzyme stability of purified A5IL97 from *E*. *coli* and *A*. *niger* was measured following the method described by [27]. The recombinant proteins were incubated at 70°C, 85°C, and 95°C in 50 mM MES at pH 6.5 for an appropriate time. The reaction mixture was further incubated with 1 mM *p*NPG at 85°C for 10 minutes and quenched with 2% Na_2_CO_3_. The end-point samples were measured for absorbance at 410 nm and normalized to zero-point incubation. The experiment was repeated twice with three biological replicates.

### Enzymatic hydrolysis of switchgrass

Thirty grams of raw putnam switchgrass was pretreated in a 1 L volume of 1-ethyl-3-methylimidazolium acetate by incubating at 160°C for 3 hours, followed by dispensing into 96 wells using a Biomek NX^P^ Lab Automation Workstation (Beckman Coulter, Carlsbad, CA). After centrifuging at 4000 rpm for 5 minutes, the biomass was recovered by adding an equal volume of water and incubating at 37°C for 1 hour while shaking at 150 rpm. The biomass was further washed with water to completely remove the IL and autoclaved at 121°C for 20 minutes. The process of biomass incubation, washing, and autoclaving was repeated two times, and the IL-free washed biomass was stored in water at 4°C until used for saccharification.

To compare saccharification efficiency of A5IL97 produced from either *E*. *coli* or *A*. *niger*, an IL-tolerant cellulase cocktail, called JTherm was used [10,12]. JTherm consists of three major components: 1) the β-glucosidase A5IL97 from *Thermotoga petrophila*, 2) a truncated cellobiohydrolase CelB from *Caldicellulosiruptor saccharolyticus* (Csac), and 3) an endoglucanase mixture from a switchgrass-adapted microbial consortia grown on microcrystalline cellulose (McCel) [10–12,28]. McCel also has xylanase activity, and can hydrolyze xylan into xylose. The recombinant enzymes were expressed in *E*. *coli* or *A*. *niger* and quantified using the Bradford method. Total 10 mg protein per g glucan in biomass was set in a final volume of 0.3 mL containing 1 mg of IL-pretreated switchgrass, with a composition of approximately 0.6 mg of glucan and 0.08 mg of xylan. Saccharification was performed by mixing a 0.15 mL volume of recombinant purified enzymes at the aforementioned enzyme loading with 1 mg of IL-pretreated switchgrass suspended in 0.15 mL of water and incubating at 70°C for up to 72 hours with constant rotating. The amount of released sugars in the hydrolysate was measured by high performance liquid chromatography (HPLC) (Agilent 1260 Infinity series, Agilent Technologies, Santa Clara, CA) with Aminex HPX-87H column (Bio-Rad, Hercules, CA) after filtration of each reaction [29]. The experiment was repeated twice with three biological replicates. A no enzyme control was conducted with IL-pretreated switchgrass incubated at 70C for 72 hrs in 50mM MES buffer pH6.5, and no increase in the initial concentration of glucose (0.01g/L) or xylose (0g/L) was detected.

### Accession numbers

All codon-optimized cellulase gene sequences were deposited in the NCBI GenBank database under accession numbers KY014107 to KY014139 (Table 1).

All *A*. *niger* strains, *E*. *coli* strains, and plasmids, used in this study are available at the Joint BioEnergy Institute under the Inventory of Composable Elements (ICE) system. All data generated or analyzed during this study are included in the manuscript and additional information.

## Results

### Expression of thermophilic bacterial cellulases in *A*. *niger*

To examine whether *A*. *niger* is a suitable host for the expression of heterologous enzymes relevant to bioenergy applications, an expression construct designed to promote secretion of heterologous enzymes was used to express 29 different bacterial cellulase genes, 3 fungal cellulases, and a GFP control in a wild-type *A*. *niger* strain (Table 1). The IL-tolerance and Thermo-tolerance of most of these enzymes have been previously reported, and are summarized in Table 1 [11,13,31]. Enzyme expression was verified by detecting cellulase activity present in culture supernatants using the model substrates *p*NPG, *p*NPC, and Azo-CMC at the optimum temperature for each enzyme. The GFP strain was used as a baseline negative control to preclude detection of any native cellulase activity, which was suppressed by cultivating strains in maltose medium. Most transformants produced detectable levels of the expected cellulase activity, confirming the successful expression and secretion of the heterologous enzymes (Table 1). Strains that produced the highest range of detected enzymatic activity include Cel9A, J30, Csac, J36, and J26 strains for the endoglucanases, and A5IL97, J14, J20, J07, and J06 strains for the β-glucosidases. Only one positively integrated gene, J11, failed to produce detectable enzymatic activity, indicating that the enzyme was either not expressed, not secreted, or secreted but not active.

### Functional comparison of a cellulase expressed in *E*. *coli* versus *A*. *niger*

**Fig 1:**
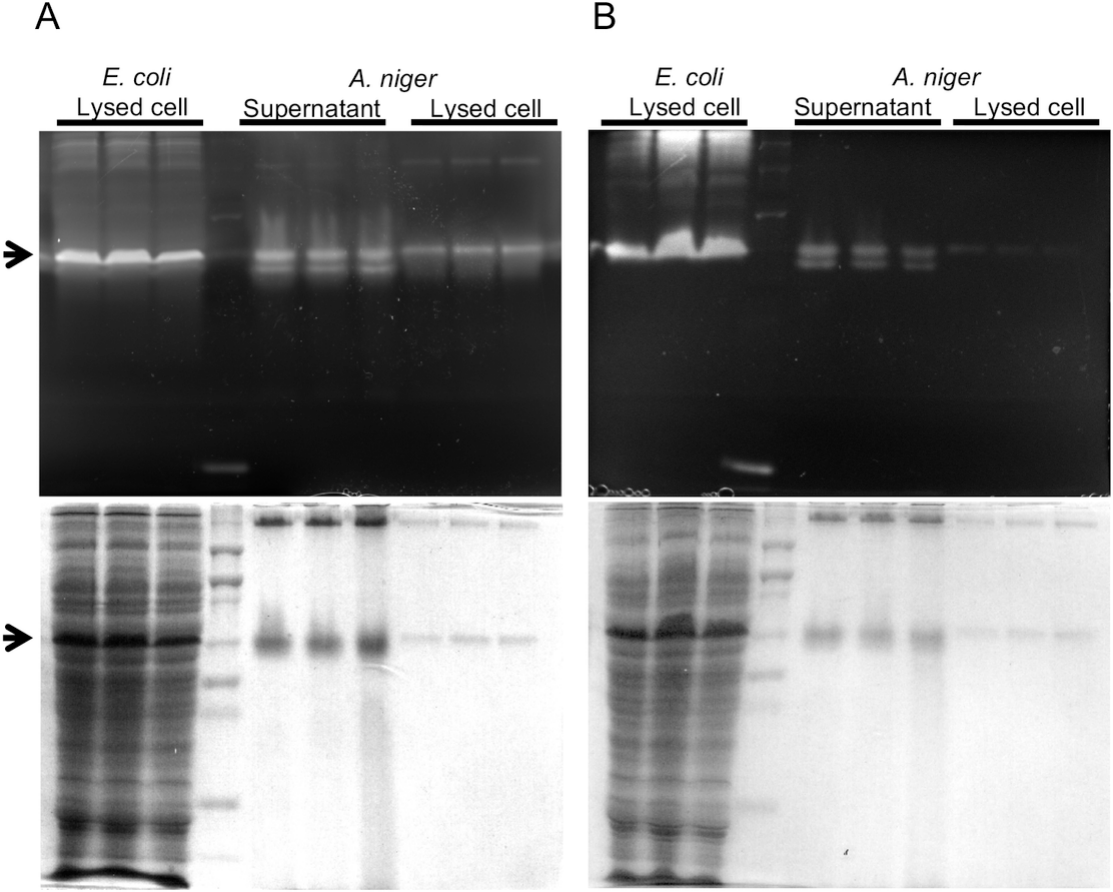
Zymography of A5IL97 produced in *E*. *coli* and *A*. *niger*. Zymography was performed with unpurified, non-denatured extracts of A5IL97, expressing strains of *E*. *coli* (Ec-A5IL97) and *A*. *niger* (An-A5IL97). Both β-glucosidase and cellobiohydrolase activities were analyzed on native gels, containing A) 4-methylumbelliferyl β-D-glucopyranoside (MUG) or B) 4-methylumbelliferyl β-D-cellobioside (MUC), respectively (top). The same gel was stained with Coomassie blue G250 after the zymography (bottom). Black arrows indicate the position of A5IL97.

To better evaluate heterologous enzyme expression in *A*. *niger*, the enzyme that exhibited the highest level of secreted β-glucosidase activity, A5IL97, was selected to be characterized in more detail. To determine if expression in *A*. *niger* impacts enzyme function, enzymatic activity of A5IL97 produced in *A*. *niger* (An-A5IL97) was compared to a version expressed in *E*. *coli* (Ec-A5IL97). The two proteins were examined on native PAGE gels used for Coomassie staining or for zymography using two model substrates to test for β-glucosidase (MUG) and cellobiohydrolase (MUC), respectively. Results show that the two enzymes had similar molecular weights and enzymatic activity, indicating that the enzyme is functionally equivalent when expressed in these two distinct hosts (Fig 1).

To further characterize A5IL97 enzyme activity, the jSALSA enzyme screening platform was utilized to determine temperature, pH and IL-tolerance ([C_2_C_1_lm][OAc]) optimums, using *p*NPG/C to monitor β-glucosidase/cellobiohydrolase activity. The jSALSA platform requires purified enzyme, so to enable purification, a new *A*. *niger* A5IL97 expression construct was built with a single histidine affinity tag. This strain, A5IL97-8×His, made it possible to purify A5IL97 from both the supernatant (13.83 mg/L) and cell pellet (7.4 mg/L), showing that 65% of the protein is secreted after 7 days of incubation (Table 2).

**Table 2:** A5IL97 production in *A*. *niger*.

	intracellular	extracellular
Wet Biomass (g/L)	99.73±6.96	
Dry Biomass (g/L)	28.53±1.21	
A5IL97 Enzyme Production (U/L)	1143.07±97.76	2032.81±425.76
A5IL97 Protein Production (mg/L)	7.40±0.17	13.83±2.42
A5IL97 Production per Wet Biomass Weight (mg/L/g WW)	73.82±1.74	137.93±24.09
A5IL97 Production per Dry Biomass Weight (mg/L/g DW)	252.69±5.97	472.17±82.48

Protein production data of a His-tagged A5IL97 produced by *A*. *niger* in a 50 mL culture. The biomass weight, enzyme, and protein production were measured after 7 days of cultivation. The numbers represent the mean of each measurement with standard deviations. The experiment was performed two times with three replicates.

**Fig 2:**
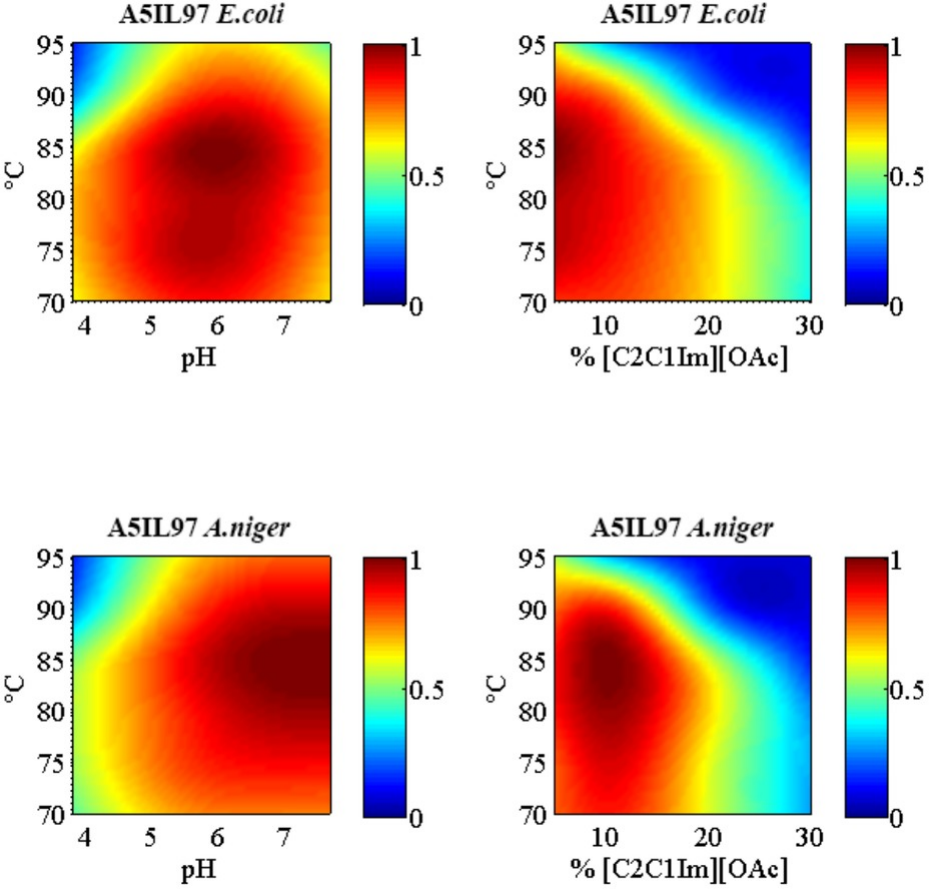
Enzymatic activity profile of A5IL97 from *E*. *coli* and *A*. *niger*. Enzyme activity profiles were generated using purified Ec-A5IL97 and An-A5IL97 at temperatures between 70 and 95°C, pH between 4 and 8, and [C_2_C_1_lm][OAc] IL concentrations between 5 and 30% (v/v). The colorimetric substrate, *p*NPG, was used to measure the enzyme activity, represented in the color bars in the legend.

A comparison of His-Tagged Ec-A5IL97 and An-A5IL97 enzymatic activates was conducted over a range of temperature, pH, and IL concentrations and A5IL97 from both organisms demonstrated moderate activity over a broad range of these conditions (Fig 2).

The optimal temperature for both enzymes was approximately 85°C. The optimal pH for the An-A5IL97 was 6.5–7.5, while Ec-A5IL97 had a slightly lower optimum around pH 6. There were also minor differences in IL-tolerance, with An-A5IL97 having slightly higher activity relative to Ec-A5IL97 at lower IL concentrations (10%) and slightly lower tolerance at higher IL concentrations (20%). However, the overall differences detected were fairly minor relative to other enzymes previously screened on the jSALSA platform, and indicate that the two enzymes are functionally equivalent.

**Fig 3:**
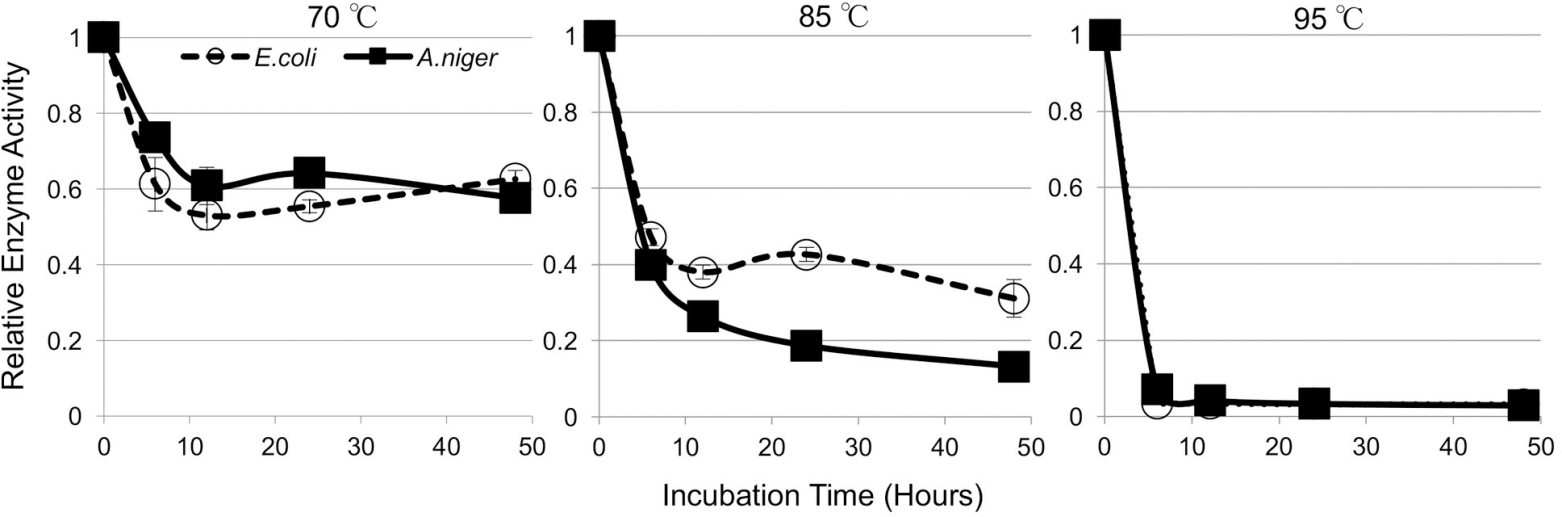
Thermostability of A5IL97 in *E*. *coli* and *A*. *niger*. Enzyme stability of A5IL97 using equal amounts of purified A5IL97 (200 μg/mL) from *E*. *coli* (Ec-A5IL97) and *A*. *niger* (An-A5IL97) at 70°C, 85°C, and 95°C. Error bars indicate the standard deviations from three replicates.

Enzyme stability is an important characteristic of cellulases used in hydrolysis of lignocellulosic biomass, and therefore the high temperature stability of A5IL97 was also examined. To characterize the thermal stability of A5IL97 from *E*. *coli* and *A*. *niger*, the purified enzymes were incubated at 0, 6, 12, 24, and 48 hours at 70°C, 85°C, and 95°C. The residual activity was then measured at the enzyme’s optimal temperature (Fig 3).

Ec-A5IL97 and An-A5IL97 had decreased residual activity in the first six hours (60% at 70°C and 40% at 85°C), but after that initial time-span both Ec-A5IL97 and An-A5IL97 appeared to stabilize at 70°C for up to 48 hours. At 85°C, the Ec-A5IL97 actually exhibited a slightly higher thermal stability after the initial dip in stability at earlier time points. Both enzymes showed a dramatic drop in stability with almost no activity observed at 95°C after the first few hours. However, the overall trends of thermal stability of Ec-A5IL97 and An-A5IL97 were similar.

**Fig 4:**
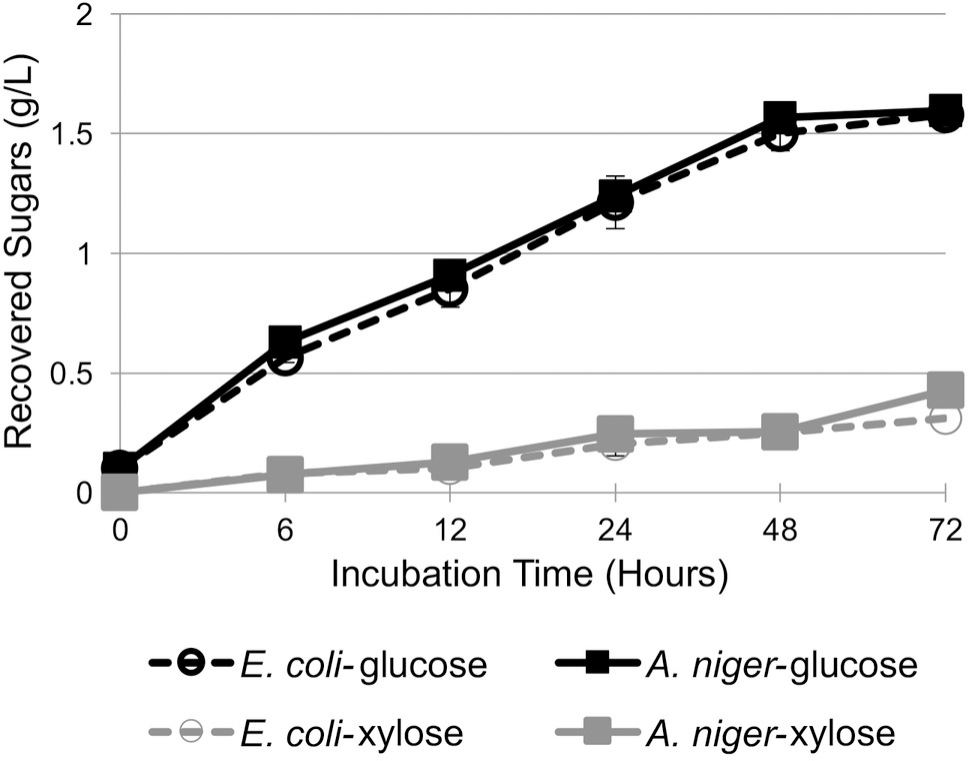
Saccharification of biomass using cellulase mixture containing A5IL97 from either *E*. *coli* or *A*. *niger*. Enzyme hydrolysis of IL-pretreated switchgrass using the JTherm cellulase mixture supplemented with either purified Ec-A5IL97 or An-A5IL97. The IL-pretreated switchgrass was washed free of IL prior to saccharification. Equivalent amounts of glucose and xylose were liberated over 72 hours. Error bars indicate the standard deviations from three replicates.

To understand how these minor functional differences between A5IL97 expressed in *E*. *coli* and *A*. *niger* impact its performance with regards to hydrolysis of lignocellulose, each version of the enzyme was substituted into a thermophilic IL-tolerant cellulase mixture developed by Park *et*. *al*., called JTherm [12], and used to hydrolyze IL-pretreated switchgrass over 72 hours at 70°C (Fig 4). A5IL97 supplies the β-glucosidase activity in the JTherm mixture, and was originally produced in *E*. *coli*. If this enzyme has the same activity when expressed in *A*. *niger*, then the sugar yields using the two different mixtures should be the same, and that is what was observed.

JTherm mixtures containing A5IL97 from either *E*. *coli* or *A*. *niger* liberated equivalent amounts of glucose and xylose over the course of the saccharification reaction. Maximum levels of glucose were reached by 48 hours, while xylose concentrations increased slightly from 48 to 72 hours. Approximately 71.8% and 72.6% of glucan was hydrolyzed to glucose by either Ec-A5IL97 or An-A5IL97 JTherm cocktails, respectively. This analysis indicates that the minor differences in enzyme activity detected when A5IL97 was expressed in *E*. *coli* or *A*. *niger* do not impact the overall performance of this enzyme within a cellulase mixture, giving further evidence that *A*. *niger* is a good production host for enzymes first characterized in *E*. *coli*

## Discussion

A number of thermophilic IL-tolerant bacterial cellulases have been previously identified for their ability to hydrolyze lignocellulose [10,13,30]. However, to be used for commercial applications, these enzymes would have to be produced at higher titers in an industrially compatible host. One of the major attractive features of *A*. *niger* is that it is currently used in industry and has been demonstrated to produce high levels of enzymes [32,33]. These attractive features were why this organism was selected and vetted as a heterologous enzyme expression host in this study.

There are known enzyme hyper-production strains of *A*. *niger* that secrete high levels of enzymes (for example, over 25 g/L of glucoamylase [32,33]), compared to the methylotrophic yeast, *Hansenula polymorpha*, that was only able to secrete 1.4 g/L of the equivalent enzyme [34]. However, many of these strains are not accessible for academic research, or their hyper-production phenotype is not mechanistically understood. Therefore, we decided to use an unmodified *A*. *niger* strain for our initial heterologous enzyme screening efforts. This provides not only a clearly understandable baseline for enzyme production, but also an opportunity to carefully follow lineages of these strains engineered and mutagenized to produce higher enzyme titers and build a mechanistically well understood “road map” of host modifications required to convert wild-type *A*. *niger* into an enzyme hyper-producer. That long-term goal will be the target of future studies using the enzymes described in this study.

Here, we demonstrated that of thirty-two different thermostable and IL-tolerant enzymes with several potential industrial applications can be successfully expressed *A*. *niger*, indicting this organisms’ clear potential as a heterologous enzyme production host. Of these enzymes, the highest enzymatic activity detected was from the strain producing A5IL97, a GH1 family β-glucosidase, producing over 20 mg/L of total heterologous enzyme (Table 2). This titer is much lower than that of strains optimized to produce high levels of native enzymes, such as glucoamylase, which is not all that surprising considering that this strain has not been engineered for optimal protein production [32,33]. The lower heterologous protein titer could be due to several reasons, including poor transcription, translation, protein folding, and protease degradation. Results of extracellular A5IL97 production in Table 2 indicated a broad range of standard deviations, compared to those of intracellular samples, suggesting the possible involvement of proteases in diminished secreted protein titers. Several extracellular proteases [35–37] are known in *A*. *niger* and the strain used in this study is known to produce them. Therefore, one approach to improve heterologous protein production would be to genetically engineer it to reduce the extracellular protease activity. For example, the fungus, *Chrysosporium lucknowense*, was genetically engineered for reduced protease production to achieve up to 100 g/L heterologous enzyme production [38]. In addition to protease activity, several studies have shown that protein secretion can be enhanced through genetic engineering to modify the unfolded protein response [39] or endoplasmic reticulum (ER) associated degradation [40,41], indicating that further genetic engineering is a promising avenue to increase heterologous enzyme production.

One interesting observation was that the three fungal cellulases, BGL1 (β-glucosidase from *Thermoascus aurantiacus*), CBH1 (cellobiohydrolase from *Trichoderma reesei*), and EG1 (endoglucanase from *Thermoascus aurantiacus*) showed low levels of enzyme expression relative to the bacterial cellulases, indicating that a close phylogenetic relationship of the source heterologous enzyme to the production host is not necessarily predictive of good expression in *A*. *niger*. Another interesting observation was that the cellulases that expressed well in *E*. *coli* [13], also expressed well in the *A*. *niger*, indicating that it may be possible to use *E*. *coli* as a system to rapidly predict whether a heterologous enzyme will express well in *A*. *niger*. This may be a very valuable tool, especially if *A*. *niger* can be engineered to eventually produce g/L quantities of heterologous enzymes.

## Conclusions

The filamentous fungus *A*. *niger* has been used to produce a broad range of native and recombinant enzymes at an industrial scale. It is able to secrete these enzymes, which is advantageous compared to other conventional laboratory organisms that require cell lysis to liberate the protein. Results in this study indicate that *A*. *niger* does indeed appear to be a good potential heterologous enzyme production host. Successful expression of a large set of heterologous enzymes is just a first step in the process of host development. Considering the high titers of native enzymes this organism has been shown to produce, there is a good possibility that *A*. *niger* will be able to produce more commercially relevant titers of heterologous enzymes through process optimization and genetic engineering.

## Supporting information

S1 Fig Thermophilic cellulase expression shuttle vector in *A*. *niger*. The plasmid, pCB1004-*glaA* contains *A*. *niger glaA* promoter, *PglaA* with the signal sequence and propeptide and *A*. *nidulans trpC* terminator, *TtrpC*. Hygromycin B is a marker for fungal transformation. Chloramphenicol is a marker for bacterial transformation. Thermophilic cellulase encoding genes were inserted to enzyme gene location (grey arrow) between restriction enzymes, *Sal*I and *Xba*I, and introduced to wild type, *A*. *niger* ATCC11414 strain. Total size of the plasmid without enzyme gene is 6.3 kb.
(TIF)

S2 Fig Detailed sequence information about the plasmid, pCB1004-*glaA*. Black bold: restriction sites, *Xho*I, *Sal*I, *Xba*I, and *Not*I from the beginning, Red: *A*. *niger glaA* promoter, Blue: *glaA s*ignal peptide, Orange: *glaA* propeptide, Black: Insert cloning site; *Sal*I-Spacer-*Xba*I, Purple: TEV protease site *A*. *niger* codon optimized (ENLYFQG), Green: Strep-His double affinity tag, Grey: *A*. *nidulans trpC* terminator, including third frame stop codon (grey underlined). Red underline indicates the primer sites, SAC001 and 004 for the fungal mutant screening. Italicized ATG with red show the start codon of *glaA* ORF.
(TIF)

S3 Fig Southern blot hybridization for A5IL97 in *A*. *niger*. A) Schematic diagram of A5IL97 fungal mutant Southern blot hybridization. Two different restriction enzymes, *Ssp*I (S) and *Dra*III (D) were utilized to digest genomic DNA of wild type ATCC11414 (WT) and hyper cellulase producing mutant, A5IL97. B) Southern blot hybridization was performed. The A5IL97 strain with *Ssp*I digestion indicate a wild type 8.5kb fragment with 5.4 kb of A5IL97 fragment. The same strain with *Dra*III digestion shows a wild type, 4.7 kb fragment as well as 2.7 kb and another size fragments, shown as asterisk of A5IL97 insertions.
(TIF)

S4 Fig A5IL97 Protein Purification. Both (A) fungal and (B) bacterial A5IL97 proteins were purified with histidine affinity purification tag. The collected flow-through (FT), washing, and elution fractions were loaded to SDS-PAGE and stained with Coomassie Blue G-250. Black arrows show the expected position of A5IL97, 51kDa.
(TIF)

## References

[1] Menon V, Rao M. Trends in bioconversion of lignocellulose: Biofuels, platform chemicals & biorefinery concept. Progress in Energy and Combustion Science. 2012 pp. 522–550. doi: 10.1016/j.pecs.2012.02.002

[2] Langholtz MH, Stokes BJ, Eaton LM. U.S. Department of Energy. 2016 Billion-Ton Report: Advancing Domestic Resources for a Thriving Bioeconomy, Vol. 1: Economic Availability of Feedstocks. Oak Ridge, TN; 2016. 10.2172/1271651

[3] Kocar G, Civas N. An overview of biofuels from energy crops: Current status and future prospects. Renew Sustain Energy Rev. 2013;28: 900–916. doi: 10.1016/j.rser.2013.08.022

[4] Caspeta L, Buijs NAA, Nielsen J. The role of biofuels in the future energy supply. Energy Environ Sci. 2013;6: 1077–1082. doi: 10.1039/c3ee24403b

[5] Klein-Marcuschamer D, Oleskowicz-Popiel P, Simmons BA, Blanch HW. The challenge of enzyme cost in the production of lignocellulosic biofuels. Biotechnol Bioeng. 2012;109: 1083–1087. doi: 10.1002/bit.24370 22095526 10.1002/bit.24370

[6] Naik SN, Goud VV, Rout PK, Dalai AK. Production of first and second generation biofuels: A comprehensive review. Renewable and Sustainable Energy Reviews. 2010 pp. 578–597. doi: 10.1016/j.rser.2009.10.003

[7] Dadi AP, Varanasi S, Schall CA. Enhancement of cellulose saccharification kinetics using an ionic liquid pretreatment step. Biotechnol Bioeng. 2006;95: 904–910. doi: 10.1002/bit.21047 16917949 10.1002/bit.21047

[8] Zhao H, Baker GA, Cowins JV. Fast enzymatic saccharification of switchgrass after pretreatment with ionic liquids. Biotechnol Prog. 2010;26: 127–133. doi: 10.1002/btpr.331 19918908 10.1002/btpr.331

[9] Shi J, Gladden JM, Sathitsuksanoh N, Kambam P, Sandoval L, Mitra D, et al One-pot ionic liquid pretreatment and saccharification of switchgrass. Green Chem. 2013;15: 2579–2589. doi: 10.1039/c3gc40545a

[10] Gladden JM, Allgaier M, Miller CS, Hazen TC, VanderGheynst JS, Hugenholtz P, et al Glycoside hydrolase activities of thermophilic bacterial consortia adapted to switchgrass. Appl Environ Microbiol. 2011;77: 5804–5812. doi: 10.1128/AEM.00032-11 21724886 10.1128/AEM.00032-11 PMC3165268

[11] Datta S, Holmes B, Park JI, Chen Z, Dibble DC, Hadi M, et al Ionic liquid tolerant hyperthermophilic cellulases for biomass pretreatment and hydrolysis. Green Chem. 2010;12: 338–345. doi: 10.1039/b916564a

[12] Park JI, Steen EJ, Burd H, Evans SS, Redding-Johnson AM, Batth T, et al A thermophilic ionic liquid-tolerant cellulase cocktail for the production of cellulosic biofuels. PLoS One. 2012;7: e37010 doi: 10.1371/journal.pone.0037010 22649505 10.1371/journal.pone.0037010 PMC3359315

[13] Gladden JM, Park JI, Bergmann J, Reyes-Ortiz V, D’haeseleer P, Quirino BF, et al Discovery and characterization of ionic liquid-tolerant thermophilic cellulases from a swithchgrass-adapted microbial community. Biotechnol Biofuels. 2014;7: 15 doi: 10.1186/1754-6834-7-15 24479406 10.1186/1754-6834-7-15 PMC3923250

[14] Nevalainen KMH, Te’o VSJ, Bergquist PL. Heterologous protein expression in filamentous fungi. Trends in Biotechnology. 2005 pp. 468–474. doi: 10.1016/j.tibtech.2005.06.002 15967521 10.1016/j.tibtech.2005.06.002

[15] Rose SH, Van Zyl WH. Exploitation of *Aspergillus niger* for the heterologous production of cellulases and hemicellulases. Open Biotechnol J. 2008;2: 167–175. doi: 10.2174/1874070700802010167

[16] Nieves RA, Ehrman CI, Adney WS, Elander RT, Himmel ME. Technical Communication: survey and analysis of commercial cellulase preparation suitable for biomass conversion to ethanol. World J Microbiol Biotechnol. 1998;14: 301–304. doi: 10.1023/A:1008871205580

[17] Fleiner A, Dersch P. Expression and export: Recombinant protein production systems for *Aspergillus*. Appl Microbiol Biotechnol. 2010 pp. 1255–1270. doi: 10.1007/s00253-010-2672-6 20532762 10.1007/s00253-010-2672-6

[18] Nevalainen H, Peterson R. Making recombinant proteins in filamentous fungi- Are we expecting too much? Front Microbiol. 2014 pp. 1–10. doi: 10.3389/fmicb.2014.00001 24578701 10.3389/fmicb.2014.00075 PMC3936196

[19] Lubertozzi D, Keasling JD. Developing *Aspergillus* as a host for heterologous expression. Biotechnology Advances. 2009 pp. 53–75. doi: 10.1016/j.biotechadv.2008.09.001 18840517 10.1016/j.biotechadv.2008.09.001

[20] Ward M, Lin C, Victoria DC, Fox BP, Fox JA, Wong DL, et al Characterization of humanized antibodies secreted by *Aspergillus niger*. Appl Environ Microbiol. 2004;70: 2567–2576. doi: 10.1128/AEM.70.5.2567-2576.2004 15128505 10.1128/AEM.70.5.2567-2576.2004 PMC404402

[21] Carroll AM, Sweigard JA, Valent B. Improved vectors for selecting resistance to hygromycin. Fungal Genet Newsl. 1994;41: 22.

[22] Yelton MM, Hamer JE, Timberlake WE. Transformation of *Aspergillus nidulans* by using a trpC plasmid. Proc Natl Acad Sci U S A. 1984;81: 1470–1474. doi: 10.1073/pnas.81.5.1470 6324193 10.1073/pnas.81.5.1470 PMC344858

[23] Grover AK, David MacMurchie D, Cushley RJ. Studies on almond emulsin beta-D-glucosidase I. Isolation and characterization of a bifunctional isozyme. BBA—Enzymol. 1977;482: 98–108. doi: 10.1016/0005-2744(77)90358-8 10.1016/0005-2744(77)90358-8 861233

[24] Wingfield P. Protein precipitation using ammonium sulfate. Curr Protoc Protein Sci. 2001;Appendix 3: Appendix 3F. doi: 10.1002/0471140864.psa03fs13 18429073 10.1002/0471140864.psa03fs13 PMC4817497

[25] Peterson R, Grinyer J, Nevalainen H. Secretome of the coprophilous fungus *Doratomyces stemonitis* C8, isolated from koala feces. Appl Environ Microbiol. 2011;77: 3793–3801. doi: 10.1128/AEM.00252-11 21498763 10.1128/AEM.00252-11 PMC3127613

[26] Feldmann T, Guenther J, Tran H, Singh A, Adams PD, Simmons BA, et al. jSALSA: High-throughput cellulase activity profiling on ionic liquid pretreated biomass [Internet]. Poster session presented at: 36th Symposium on biotechnology for Fuels and Chemiclas. Clearwater Beach, FL; 2014. Available: https://sim.confex.com/sim/36th/webprogram/Paper26644.html

[27] Park JI, Kent MS, Datta S, Holmes BM, Huang Z, Simmons BA, et al Enzymatic hydrolysis of cellulose by the cellobiohydrolase domain of CelB from the hyperthermophilic bacterium *Caldicellulosiruptor saccharolyticus*. Bioresour Technol. 2011;102: 5988–5994. doi: 10.1016/j.biortech.2011.02.036 21421309 10.1016/j.biortech.2011.02.036

[28] Gladden JM, Eichorst SA, Hazen TC, Simmons BA, Singer SW. Substrate perturbation alters the glycoside hydrolase activities and community composition of switchgrass-adapted bacterial consortia. Biotechnol Bioeng. 2012;109: 1140–1145. doi: 10.1002/bit.24388 22125273 10.1002/bit.24388

[29] Hiras J, Wu YW, Deng K, Nicora CD, Aldrich JT, Frey D, et al Comparative community proteomics demonstrates the unexpected importance of actinobacterial glycoside hydrolase family 12 protein for crystalline cellulose hydrolysis. MBio. 2016;7 doi: 10.1128/mBio.01106-16 27555310 10.1128/mBio.01106-16 PMC4999548

[30] D’haeseleer P, Gladden JM, Allgaier M, Chain PSG, Tringe SG, Malfatti SA, et al Proteogenomic analysis of a thermophilic bacterial consortium adapted to deconstruct switchgrass. PLoS One. 2013;8 doi: 10.1371/journal.pone.0068465 23894306 10.1371/journal.pone.0068465 PMC3716776

[31] Chen Z, Pereira JH, Liu H, Tran HM, Hsu NSY, Dibble D, et al Improved activity of a thermophilic cellulase, Cel5A, from *Thermotoga maritima* on ionic liquid pretreated switchgrass. PLoS One. 2013;8 doi: 10.1371/journal.pone.0079725 24244549 10.1371/journal.pone.0079725 PMC3828181

[32] Ward OP. Production of recombinant proteins by filamentous fungi. Biotechnol Adv. 2012;30: 1119–1139. doi: 10.1016/j.biotechadv.2011.09.012 21968147 10.1016/j.biotechadv.2011.09.012

[33] Ward OP, Qin WM, Dhanjoon J, Ye J, Singh A. Physiology and biotechnology of *Aspergillus*. Adv Appl Microbiol. 2005 pp. 1–75. doi: 10.1016/S0065-2164(05)58001-8 10.1016/S0065-2164(05)58001-8 16543029

[34] Gellissen G, Janowicz ZA, Merckelbach A, Piontek M, Keup P, Weydemann U, et al Heterologous gene expression in *Hansenula polymorpha*: efficient secretion of glucoamylase. Biotechnology (N Y). 1991;9: 291–295. doi: 10.1038/nbt0391-291 1367303 10.1038/nbt0391-291

[35] Budak S, Zhou M, Brouwer C, Wiebenga A, Benoit I, Di Falco M, et al A genomic survey of proteases in Aspergilli. BMC Genomics. 2014;15: 523 doi: 10.1186/1471-2164-15-523 24965873 10.1186/1471-2164-15-523 PMC4102723

[36] Pel HJ, de Winde JH, Archer DB, Dyer PS, Hofmann G, Schaap PJ, et al Genome sequencing and analysis of the versatile cell factory *Aspergillus niger* CBS 513.88. Nat Biotechnol. 2007;25: 221–31. doi: 10.1038/nbt1282 17259976 10.1038/nbt1282

[37] Mattern IE, van Noort JM, van den Berg P, Archer DB, Roberts IN, van den Hondel CAMJJ. Isolation and characterization of mutants of *Aspergillus niger* deficient in extracellular proteases. Mol Gen Genet. 1992;234: 332–336. doi: 10.1007/BF00283855 1508158 10.1007/BF00283855

[38] Demain AL, Vaishnav P. Production of recombinant proteins by microbes and higher organisms. Biotechnol Adv. 2009 pp. 297–306. doi: 10.1016/j.biotechadv.2009.01.008 19500547 10.1016/j.biotechadv.2009.01.008

[39] Guillemette T, van Peij NNME, Goosen T, Lanthaler K, Robson GD, van den Hondel C a MJJ, et al Genomic analysis of the secretion stress response in the enzyme-producing cell factory *Aspergillus niger*. BMC Genomics. 2007;8: 158 doi: 10.1186/1471-2164-8-158 17561995 10.1186/1471-2164-8-158 PMC1894978

[40] Carvalho NDSP, Arentshorst M, Kooistra R, Stam H, Sagt CM, Van Den Hondel CAMJJ, et al Effects of a defective ERAD pathway on growth and heterologous protein production in *Aspergillus niger*. Appl Microbiol Biotechnol. 2011;89: 357–373. doi: 10.1007/s00253-010-2916-5 20922374 10.1007/s00253-010-2916-5 PMC3016150

[41] Jacobs DI, Olsthoorn MMA, Maillet I, Akeroyd M, Breestraat S, Donkers S, et al Effective lead selection for improved protein production in *Aspergillus niger* based on integrated genomics. Fungal Genet Biol. 2009;46: S141–S152. doi: 10.1016/j.fgb.2008.08.012 18824119 10.1016/j.fgb.2008.08.012

