# Supplementary figures and images for: Expression of naturally ionic liquid-tolerant thermophilic cellulases in Aspergillus niger
Source: PLoS One. 2017 Dec 27;12(12):e0189604. doi: 10.1371/journal.pone.0189604 (PMC5744941; doi:10.1371/journal.pone.0189604)

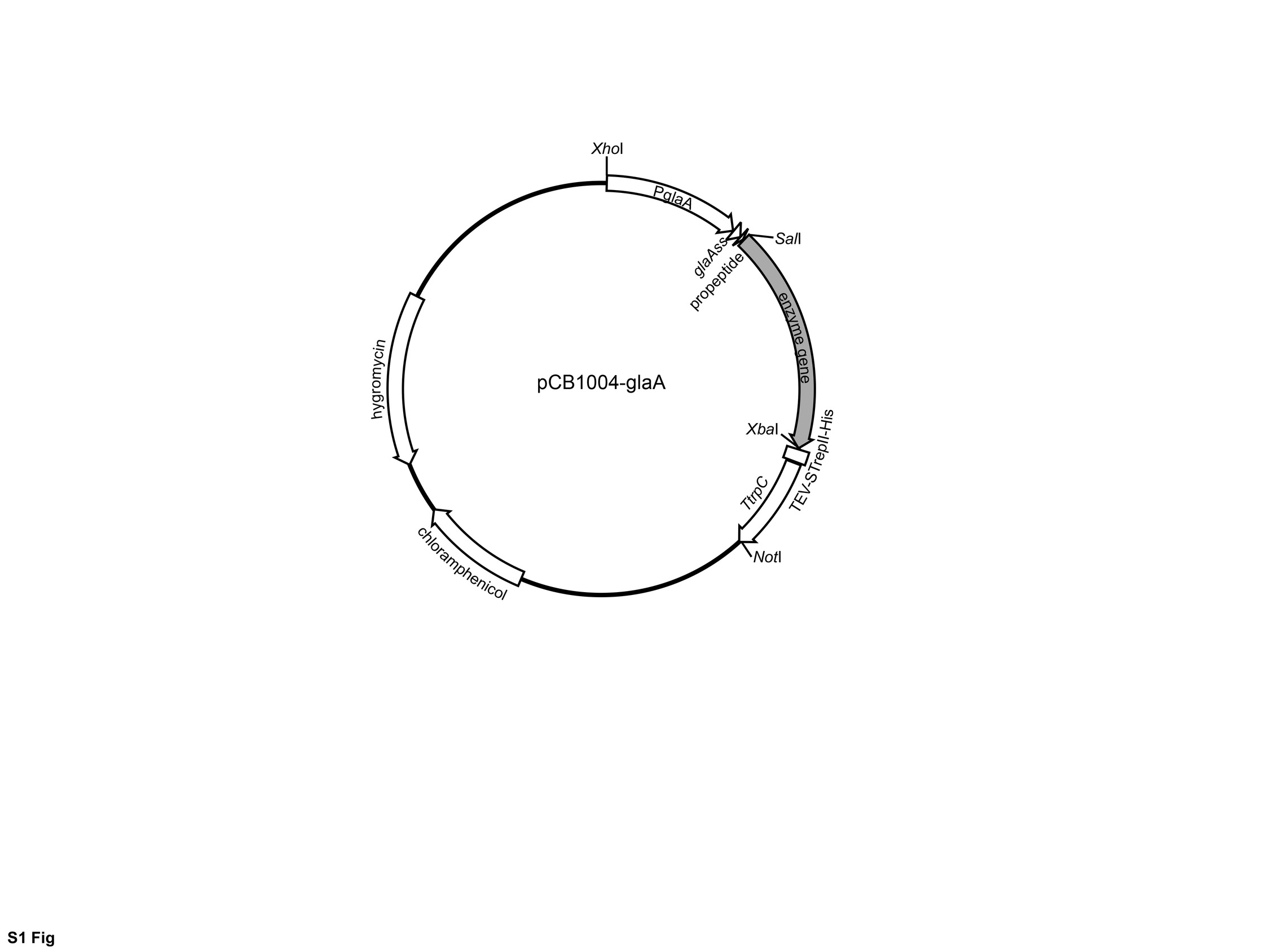

Supplement: S1 Fig — The plasmid, pCB1004-glaA contains A. niger glaA promoter, PglaA with the signal sequence and propeptide and A. nidulans trpC terminator, TtrpC. Hygromycin B is a marker for fungal transformation. Chloramphenicol is a marker for bacterial transformation. Thermophilic cellulase encoding genes were inserted to enzyme gene location (grey arrow) between restriction enzymes, SalI and XbaI, and introduced to wild type, A. niger ATCC11414 strain. Total size of the plasmid without enzyme gene is 6.3 kb. (TIF) [file pone.0189604.s001.tif]

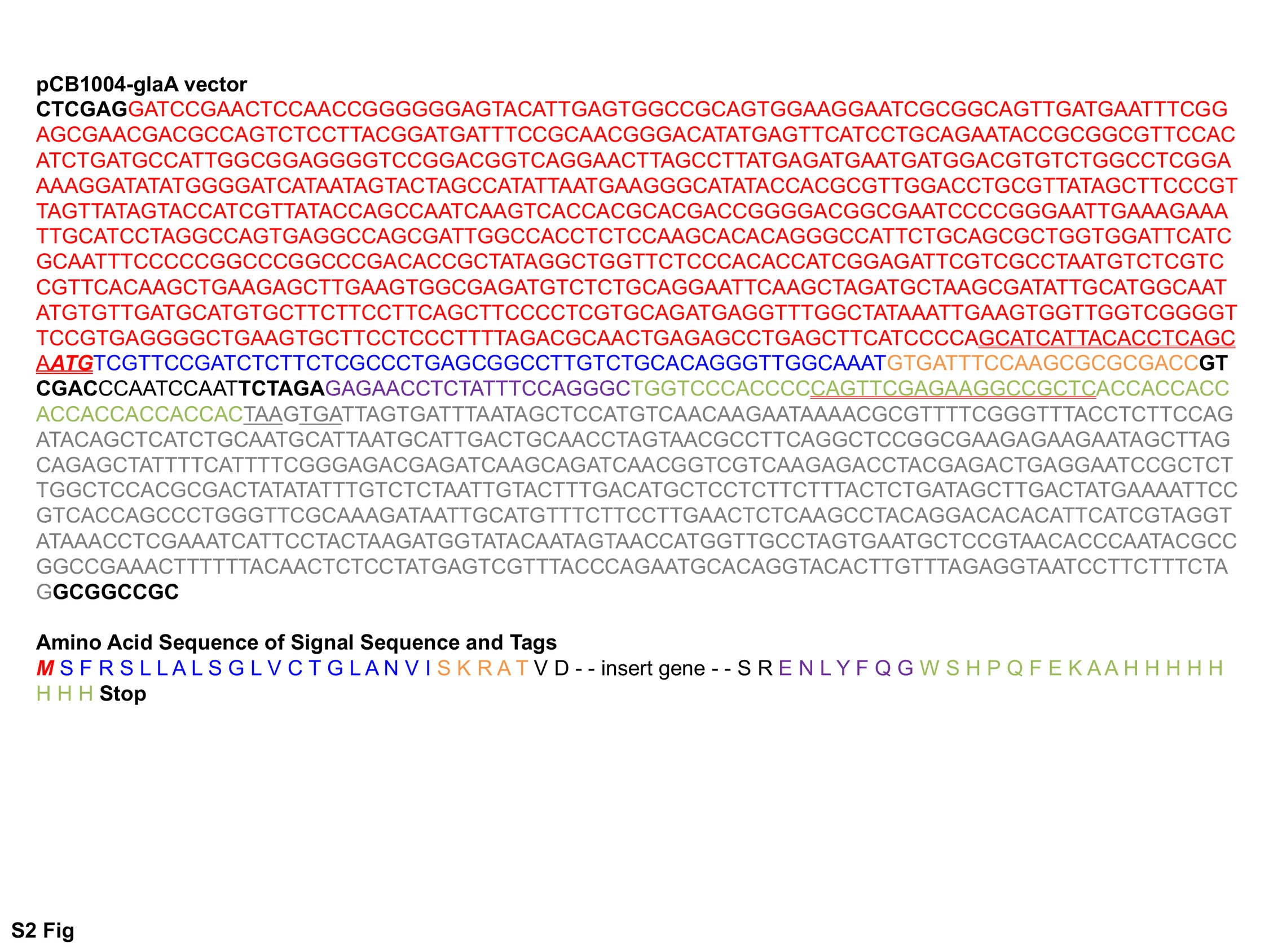

Supplement: S2 Fig — Black bold: restriction sites, XhoI, SalI, XbaI, and NotI from the beginning, Red: A. niger glaA promoter, Blue: glaA signal peptide, Orange: glaA propeptide, Black: Insert cloning site; SalI-Spacer-XbaI, Purple: TEV protease site A. niger codon optimized (ENLYFQG), Green: Strep-His double affinity tag, Grey: A. nidulans trpC terminator, including third frame stop codon (grey underlined). Red underline indicates the primer sites, SAC001 and 004 for the fungal mutant screening. Italicized ATG with red show the start codon of glaA ORF. (TIF) [file pone.0189604.s002.tif]

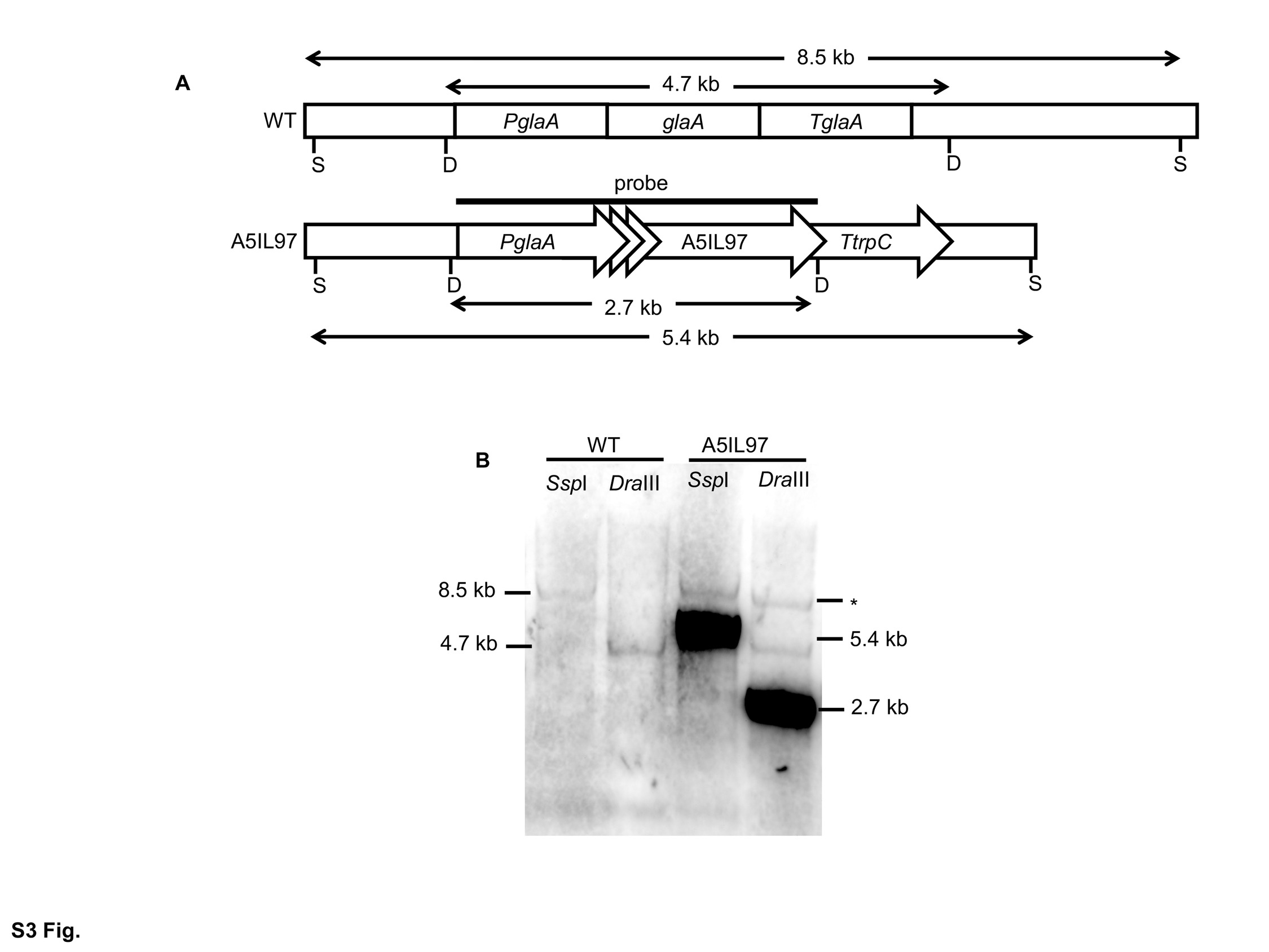

Supplement: S3 Fig — A) Schematic diagram of A5IL97 fungal mutant Southern blot hybridization. Two different restriction enzymes, SspI (S) and DraIII (D) were utilized to digest genomic DNA of wild type ATCC11414 (WT) and hyper cellulase producing mutant, A5IL97. B) Southern blot hybridization was performed. The A5IL97 strain with SspI digestion indicate a wild type 8.5kb fragment with 5.4 kb of A5IL97 fragment. The same strain with DraIII digestion shows a wild type, 4.7 kb fragment as well as 2.7 kb and another size fragments, shown as asterisk of A5IL97 insertions. (TIF) [file pone.0189604.s003.tif]

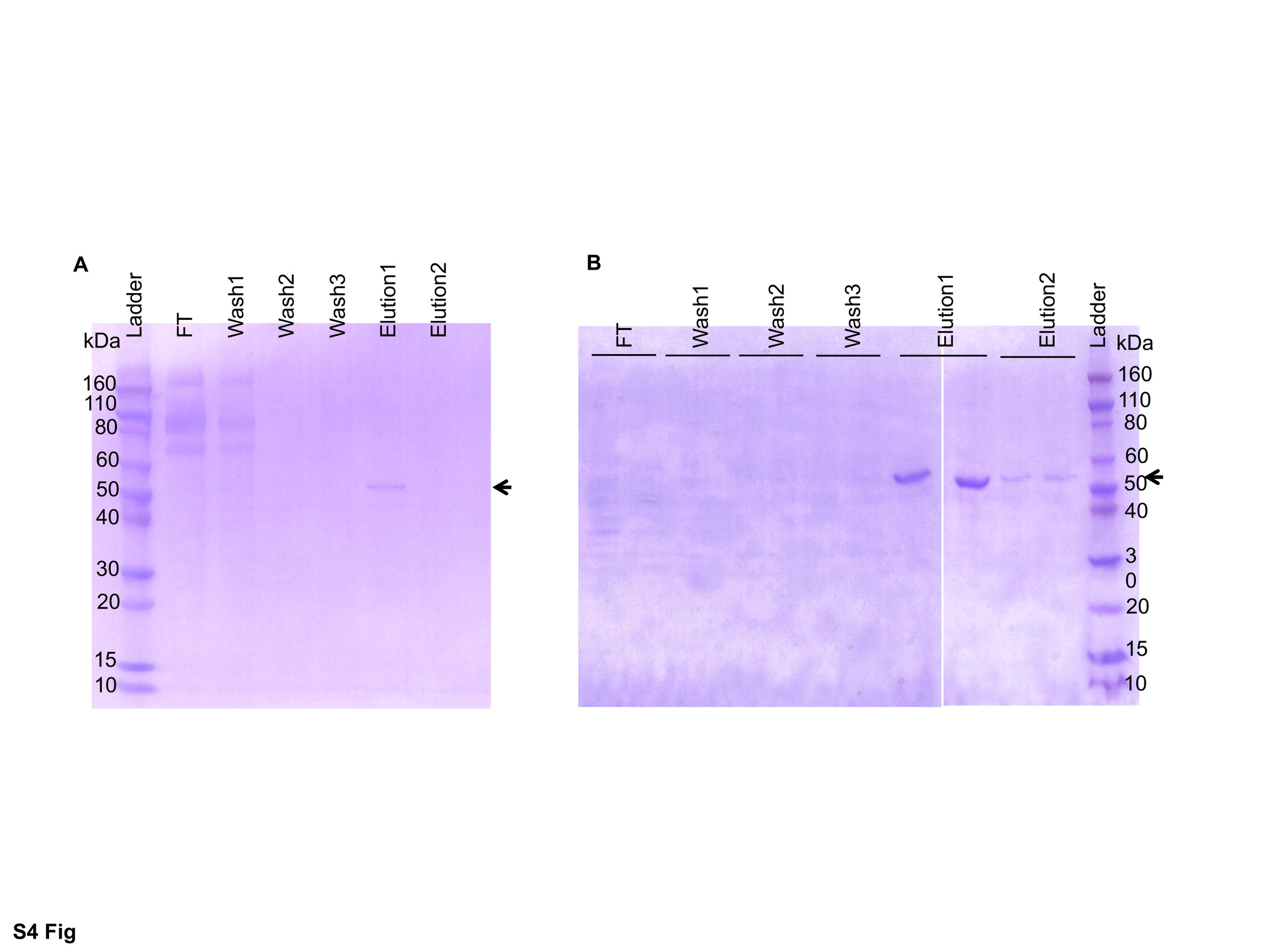

Supplement: S4 Fig — Both (A) fungal and (B) bacterial A5IL97 proteins were purified with histidine affinity purification tag. The collected flow-through (FT), washing, and elution fractions were loaded to SDS-PAGE and stained with Coomassie Blue G-250. Black arrows show the expected position of A5IL97, 51kDa. (TIF) [file pone.0189604.s004.tif]
